# Case Report: Cerebellar ALK+ ALCL: diagnostic challenges and therapeutic innovation

**DOI:** 10.3389/fonc.2026.1620819

**Published:** 2026-03-25

**Authors:** Zengfang Hao, Xuhan Wang, Zhihong Gao, Lan Yang

**Affiliations:** 1 Department of Neurology, Second Hospital of Hebei Medical University, Shijiazhuang, Hebei, China; 2 Key Laboratory of Clinical Neurology (Hebei Medical University), Ministry of Education, Shijiazhuang, Hebei, China

**Keywords:** anaplastic large cell lymphoma, ALK+, cerebellar, Ki-67 index, primary CNS lymphoma, precision oncology

## Abstract

Primary anaplastic lymphoma kinase (ALK)-positive anaplastic large cell lymphoma (ALCL) of the central nervous system (CNS) is an exceedingly rare T-cell malignancy, with only a few adult cases reported worldwide. To our knowledge, this represents the first reported adult case of cerebellar ALK+ ALCL from China, highlighting both diagnostic challenges and therapeutic innovations in neuro-oncology. We present a 35-year-old male with subacute cerebellar syndrome initially misdiagnosed as infectious encephalitis due to overlapping clinical, radiological, and cerebrospinal fluid (CSF) findings. Progressive neurological deterioration despite empiric antimicrobial therapy prompted histopathological evaluation, which confirmed ALK+ ALCL with a high Ki-67 index (60%). Targeted therapy with the CNS-penetrant ALK inhibitor alectinib induced rapid clinical improvement and significant radiographic regression. This case highlights the diagnostic pitfalls of CNS ALCL, emphasizes the necessity of early biopsy in atypical lesions, and demonstrates the transformative potential of molecularly targeted therapies for rare neuro-oncological malignancies.

## Graphical Abstract

**Figure:**
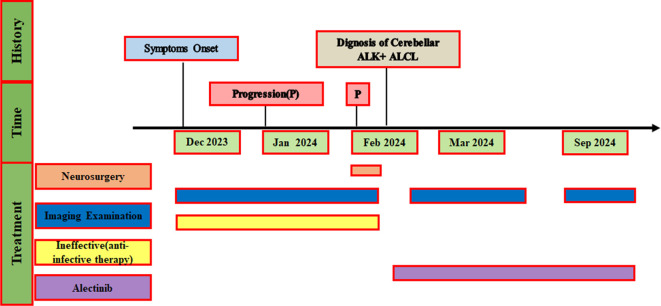
Timeline.

## Introduction

Anaplastic large cell lymphoma (ALCL), a CD30-positive T-cell neoplasm, predominantly occurs in pediatric populations (1). Primary central nervous system (CNS) involvement by ALK-positive ALCL is exceptionally rare, constituting <4% of primary CNS lymphomas (PCNSL) and typically involving supratentorial regions (2, 3). Cerebellar localization, documented in fewer than five adult cases, poses unique diagnostic challenges due to nonspecific symptoms (e.g., fever, headache, neurological deficits) and radiological mimicry of inflammatory lesions (4, 5). Cerebrospinal fluid (CSF) analysis often reveals neutrophilic pleocytosis and hyperproteinorrhachia, further misleading clinicians toward empiric antimicrobial therapy (6). Conventional PCNSL treatments (e.g., high-dose methotrexate-based regimens) exhibit limited efficacy in ALK+ ALCL, necessitating molecularly targeted strategies (7). We present a novel case of cerebellar ALK+ ALCL, emphasizing the imperative of histopathological confirmation in diagnostically challenging CNS lesions and the remarkable efficacy of alectinib—a CNS-penetrant ALK inhibitor—in this aggressive lymphoma subset.

## Case report

**Figure 1:**
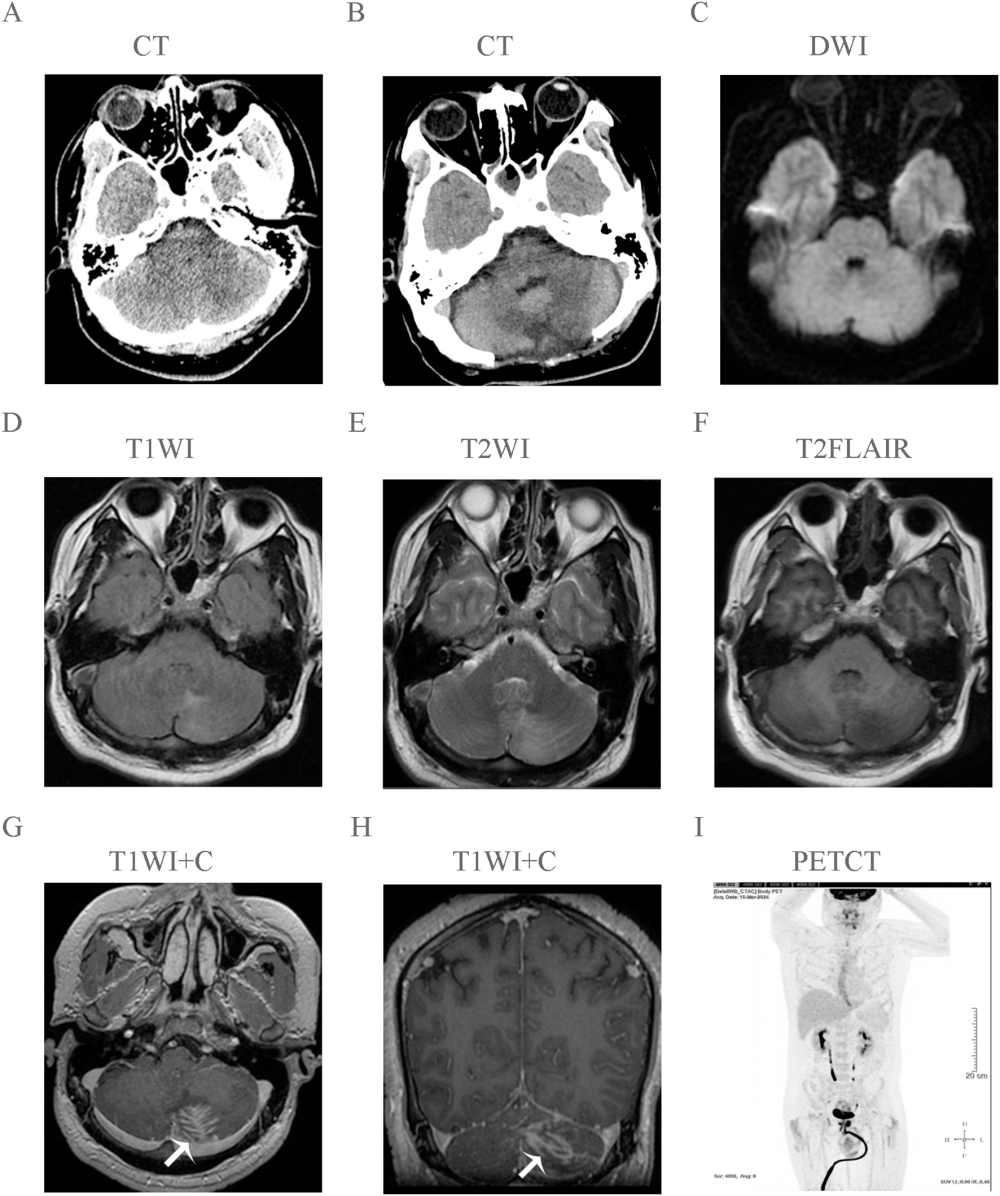
Multimodal neuroimaging of primary cerebellar ALK-positive anaplastic large cell lymphoma. **(A)** Preoperative non-contrast axial computed tomography (CT) reveals a patchy hypodense lesion in the left cerebellar hemisphere. **(B)** Postoperative non-contrast axial CT scan status after surgical resection. **(C)** Diffusion-weighted imaging (DWI) shows an isointense signal within the lesion, indicating no restricted diffusion. **(D)** Axial T1-weighted image (T1WI) demonstrates an irregular hypointense focus (arrow) in the left cerebellum. **(E)** Axial T2-weighted image (T2WI) reveals a confluent hyperintense area (arrow). **(F)** Axial T2-FLAIR image highlights perilesional hyperintensity (arrow) suggestive of vasogenic edema. **(G, H)** Axial and sagittal post-contrast T1-weighted images (T1WI+C) display delicate, linear “feather-like” enhancement patterns (arrows) at the lesion periphery. **(I)** Whole-body ^18^F-FDG PET-CT maximum intensity projection (MIP) image shows no abnormal extracranial metabolic activity, excluding systemic involvement.

A previously healthy, immunocompetent 35-year-old male with no significant medical history presented with a 25-day history of subacute fever, holocranial headache, and progressive gait instability. Initial management at a local facility included antiviral therapy for suspected influenza, without improvement. Cranial CT (January 18, 2024) revealed a hypodense lesion within the left cerebellar hemisphere (**Figure 1A**). Subsequent MRI (January 20, 2024) demonstrated T1 hypointensity, T2 hyperintensity, and a characteristic feather-like enhancement pattern on post-contrast sequences, without diffusion restriction (**Figures 1C–I**). CSF analysis (January 22, 2024) showed neutrophilic pleocytosis (white blood cell count 180×10^6^/L), markedly elevated protein (1708 mg/L), and hypoglycorrhachia (1.45 mmol/L), Stains for acid-fast bacilli, cryptococcal antigen, and India ink India ink were negative. CSF cytology demonstrated degenerated neutrophils, lymphocytes, and monocytes, with no evidence of malignant cells. The patient received empirical broad-spectrum antimicrobial therapy without clinical improvement, prompting referral to our institution for further management.

Upon admission, neurological examination revealed truncal ataxia, dysdiadochokinesia, and mild dysarthria. Serological screening for HIV, hepatitis B and C viruses, and syphilis was negative. Comprehensive autoimmune and viral serological panels were likewise unremarkable. A repeat lumbar puncture (January 25, 2024) confirmed persistent neutrophilic pleocytosis (WBC 160 × 10^6^/L), elevated protein (1120 mg/L), and hypoglycorrhachia (2.34 mmol/L). CSF PCR for Mycobacterium tuberculosis and fungal cultures were negative, and the autoantibodies related to autoimmune diseases and the common demyelinating antibodies in both serum and cerebrospinal fluid were all negative. The initial diagnostic consideration strongly favored an infectious encephalitis. However, during continued empirical anti-infective treatment, the patient experienced an abrupt neurological decline, manifesting as impaired consciousness. This acute deterioration necessitated an emergent suboccipital craniectomy and resection of the cerebellar lesion.

**Figure 2:**
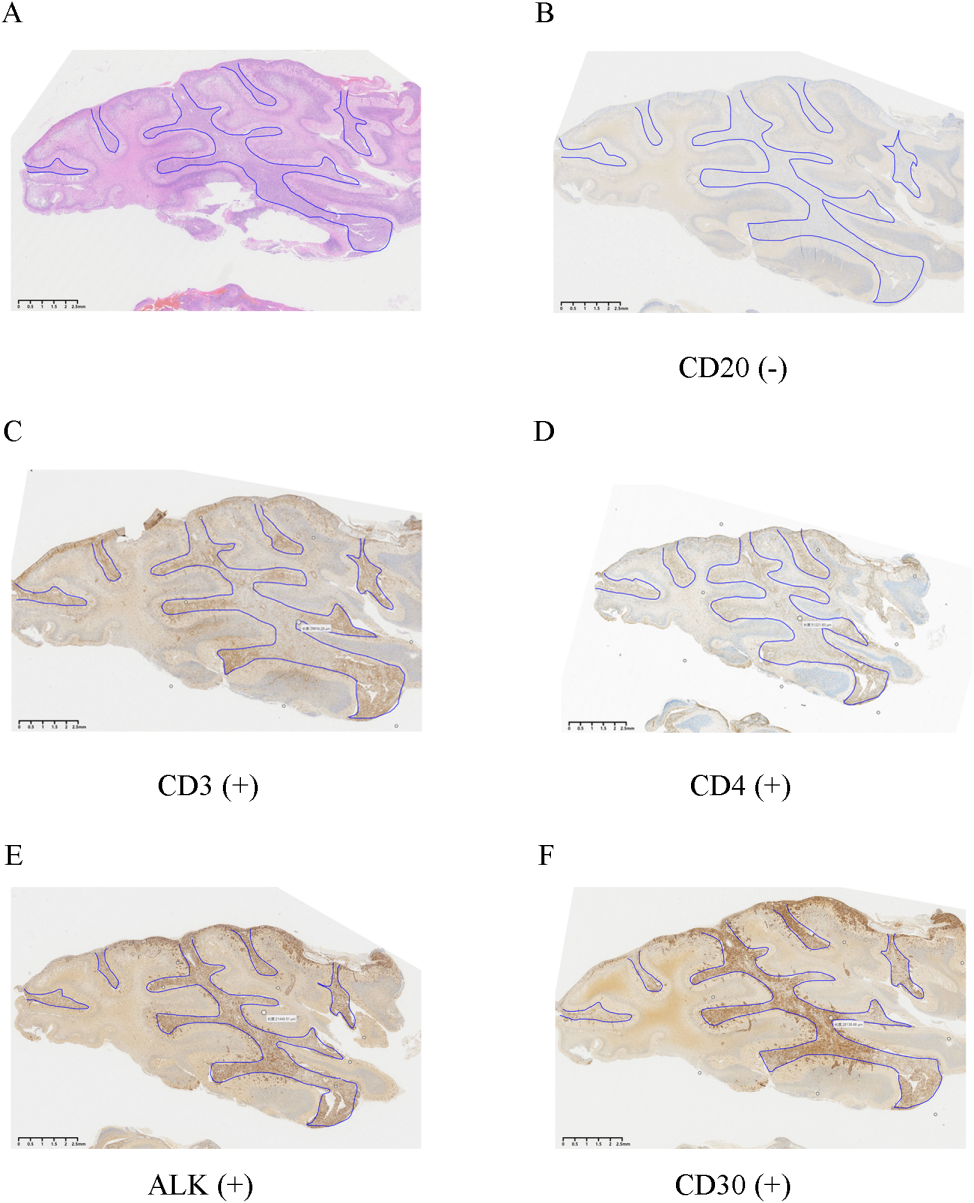
Histopathological and immunophenotypic features of cerebellar ALK-positive ALCL (low-power view). **(A)** Low-power hematoxylin and eosin (H&E) staining demonstrates monotonous lymphoid tumor cells infiltrating with an irregular interstitial growth pattern along the molecular layer of the cerebellar surface (representative area demarcated by the blue box). **(B)** Immunohistochemistry for the B-cell marker CD20 is negative (representative area demarcated by the blue box). **(C)** Tumor cells show aberrant partial positivity for CD3 (representative area demarcated by the blue box). **(D)** A subset of tumor cells is positive for CD4 (representative area demarcated by the blue box). **(E)** Tumor cells exhibit strong, diffuse cytoplasmic and membranous anaplastic lymphoma kinase (ALK) immunoreactivity (representative area demarcated by the blue box). **(F)** Robust and homogeneous CD30 positivity is observed (representative area demarcated by the blue box). Scale bar for **(A–F)**: 100 μm.

**Figure 3:**
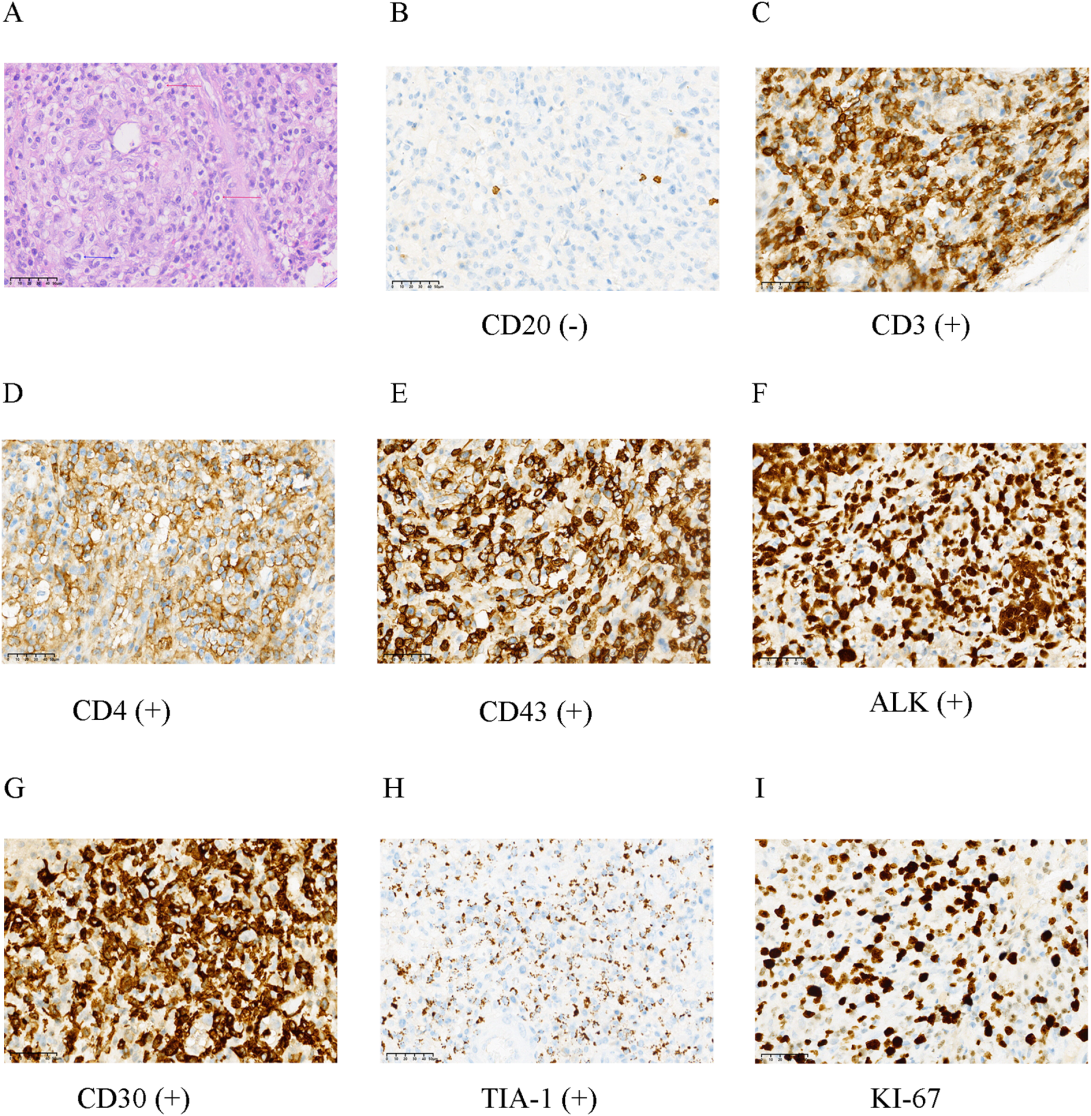
High-power cytomorphological and immunophenotypic characterization of cerebellar ALK-positive ALCL. **(A)** High-power hematoxylin and eosin (H&E) staining reveals a polymorphic tumor cell population with distinct cell membranes, abundant pale cytoplasm, and a spectrum of cytomorphology. Characteristic “fried egg”-like cells (red arrowheads) and cells with *reniform* (kidney-shaped) nuclei (blue arrowheads) are evident. **(B)** CD20 immunostaining is negative, confirming the absence of B-cell differentiation. **(C)** Tumor cells show aberrant partial positivity for CD3. **(D)** CD4 immunostaining highlights a subset of positive tumor cells. **(E)** Diffuse CD43 expression is observed. **(F)** Tumor cells exhibit strong cytoplasmic and nuclear positivity for ALK. **(G)** Robust homogeneous CD30 expression is present. **(H)** Cytoplasmic expression of T-cell intracellular antigen-1 (TIA-1) is detected. **(I)** The Ki-67 proliferation index is high (approximately 60%). Immunohistochemistry was performed using the following antibodies: ALK (clone MXR016), CD30 (clone MX080), CD3 (clone MX036), CD43 (clone MX099), TIA-1 (clone 2G9A10F5), Ki-67 (MXR002), and CD20 (clone MX003), All antibodies were obtained from Fuzhou Maixin Biotechnology Development Co., Ltd. All immunohistochemical staining was performed using an automated immunostainer according to the manufacturer’s protocols. Appropriate positive and negative controls were included for all assays. Appropriate positive and negative controls were included for all immunohistochemical assays. Scale bar for **(A–I)** 50 μm.

Histological examination revealed a diffuse, infiltrative tumor growing through the cerebellar sulci. The neoplasm was composed of large, pleomorphic lymphoid cells exhibiting hallmark reniform nuclei and prominent “fried egg” morphology, set against a background of reactive neuroglial hyperplasia. Notably, neither necrosis nor leptomeningeal involvement was identified. (**Figures 2A**, **3A**). Immunohistochemistry confirmed a diagnostic immunophenotype: the tumor cells showed strong nuclear and cytoplasmic positivity for ALK (**Figures 2E**, **3F**), membranous and Golgi-pattern expression of CD30 (**Figures 2F**, **3G**), and aberrant partial CD3 reactivity (**Figures 2C**, **3C**). A subset of the neoplastic population exhibited positivity for CD4 (**Figures 2D**, **3D**). The Ki-67 proliferation index was markedly elevated at 60% (**Figure 3I**). The neoplastic cells were negative for CD20 (**Figures 2B**, **3B**). Cytoplasmic expression of TIA-1 was observed, supporting a cytotoxic T-cell lineage (**Figure 3H**). Staining for CD43 was diffusely positive (**Figure 3E**). A subsequent whole-body ^18^F-FDG PET-CT scan revealed no evidence of systemic lymphoma involvement (**Figure 1I**), confirming the diagnosis of primary CNS ALK-positive ALCL.

Based on the molecular profile, targeted therapy with alectinib (600 mg orally, twice daily) was initiated. The treatment was well-tolerated, with no hepatotoxicity or other significant adverse events reported. Monitoring included serial neurological assessments, monthly brain MRI, and periodic liver function tests. The patient reported a significant improvement in quality of life and functional independence within two weeks of initiating alectinib. At the 6-month follow-up, CT (**Supplementary Figure 1**) demonstrated significant radiographic regression of the cerebellar lesion. The patient was asymptomatic and had achieved a full functional recovery, with a modified Rankin Scale score of 0. He expressed profound satisfaction with the rapid symptomatic resolution and restored quality of life.

## Discussion

This case exemplifies the considerable diagnostic and therapeutic complexities of primary cerebellar ALK+ ALCL, a rarity compounded by its clinicoradiological overlap with inflammatory pathologies. While supratentorial involvement dominates CNS lymphoma (6), cerebellar localization, as observed here, predisposes patients to rapid neurological decline via obstructive hydrocephalus and brainstem compression—a mechanism corroborated by prior reports (4). Notably, the lesion’s T2 hyperintensity and feather-like enhancement pattern, in the absence of diffusion restriction, initially diverted diagnostic focus toward encephalitis, a well-documented pitfall in ALCL diagnostic (8, 9). The CSF profile, characterized by neutrophilic pleocytosis and hyperproteinorrhachia, further misdirected the clinical management, delaying definitive histopathological evaluation by nearly three weeks—a critical interval that recent European Association of Neuro-Oncology (EANO) guidelines aim to shorten by advocating for early biopsy in refractory or atypical CNS lesions (10).

The initial differential diagnoses broadly included infectious encephalitis, demyelinating disease, and primary glioma. Upon histopathological examination, the differential diagnosis narrowed to neoplasms of lymphohematopoietic origin, primarily including diffuse large B-cell lymphoma (DLBCL) and other T-cell lymphomas. This case underscores that histopathological confirmation remains the diagnostic cornerstone. The immunophenotype was diagnostic: the neoplastic cells exhibited strong and diffuse co-expression of ALK and CD30, along with positivity for T-cell antigens CD4 and CD43, and an aberrant (partial) CD3 reactivity, while being negative for the B-cell marker CD20, which effectively excluded DLBCL. This profile, complemented by TIA-1 expression, confirms a cytotoxic T-cell lineage and definitively establishes the diagnosis of ALK-positive ALCL (11, 12). Unlike typical CNS lymphomas that favor supratentorial regions (1, 12), this tumor’s infiltration along the cerebellar molecular layer precipitated the rapid clinical decline. The definitive immunophenotype (ALK+/CD30+/CD3+) and high Ki-67 index (60%) not only affirmed an aggressive T-cell lineage but also suggested a biology potentially amenable to targeted inhibition. The observed high proliferative activity did not preclude a dramatic response to alectinib, consistent with the concept of “oncogene addiction” in ALK-driven malignancies, which can render them highly vulnerable to targeted therapy irrespective of the proliferation index (13).

Alectinib was selected for its superior CNS penetration compared to first-generation ALK inhibitors, attributable to its low affinity for P-glycoprotein efflux transporters and high intrinsic blood-brain barrier permeability, making it an ideal candidate for CNS lymphoma (14). This pharmacokinetic advantage is paramount in neuro-oncology, where effective drug delivery often constitutes the principal therapeutic barrier. Unlike conventional methotrexate-based chemotherapy, alectinib’s targeted mechanism and favorable CNS bioavailability enabled rapid and sustained remission in this patient, aligning with emerging evidence supporting ALK inhibitors in CNS disease.

This report also challenges traditional prognostic assumptions in CNS ALCL. Historically, a high Ki-67 index portends a poor outcome; however, the advent of molecular targeting may decouple proliferative vigor from chemoresistance. Future studies should explore the durability of response and optimal sequencing of ALK inhibitors in primary CNS lymphomas, particularly for lesions with atypical radiological signatures.

In conclusion, this case of primary cerebellar ALK+ ALCL highlights the diagnostic pitfalls of rare CNS lymphomas and the transformative promise of precision oncology. It underscores that a subacute cerebellar syndrome which progresses despite empiric antimicrobial therapy, particularly when associated with feather-like enhancement on MRI and marked CSF hyperproteinorrhachia, should raise a high suspicion for CNS lymphoma and prompt consideration for early biopsy. Therefore, clinicians must maintain a high index of suspicion for lymphoma in atypical cerebellar lesions, prioritizing early biopsy in cases refractory to empirical therapy. The profound efficacy of alectinib in this context underscores the imperative to integrate molecular profiling into standard neuro-oncological practice, redefining prognostic expectations for ALK-driven CNS malignancies.

## Limitations

Several limitations of this report warrant acknowledgment: (1) its nature as a single case report with intermediate-term follow-up; (2) the absence of molecular characterization of the specific ALK fusion variant; (3) the lack of additional immunophenotyping (e.g., CD4/CD8) and T-cell receptor clonality analysis due to the patient’s refusal to provide additional consent for these supplementary studies the patient refuse; and (4) the unavailability of pre-treatment advanced neuroimaging sequences (e.g., perfusion MRI) for correlation with treatment response.

## Data Availability

The datasets presented in this study can be found in online repositories. The names of the repository/repositories and accession number(s) can be found in the article/**Supplementary Material**.

## References

[1] Stein H Foss HD Durkop H Marafioti T Delsol G Pulford K . CD30(+) anaplastic large cell lymphoma: a review of its histopathologic, genetic, and clinical features. Blood. (2000) 96:3681–3695. doi: 10.1182/blood.V96.12.3681, PMID: 11090048

[2] Menon MP Nicolae A Meeker H Raffeld M Xi L Jegalian AG . Primary CNS T-cell lymphomas: A clinical, morphologic, immunophenotypic, and molecular analysis. Am J Surg Pathol. (2015) 39:1719–1729. doi: 10.1097/PAS.0000000000000503, PMID: 26379152 PMC4644095

[3] Bataille B Delwail V Menet E Gandermann P Ingrand P Wagner M . Primary intracerebral Malignant lymphoma: report of 248 cases. J Neurosurg. (2000) 92:261–266. doi: 10.3171/jns.2000.92.2.0261, PMID: 10659013

[4] Geetha N Sreelesh KP Nair R Mathews A . Anaplastic large cell lymphoma presenting as a cerebellar mass. Hematol Oncol Stem Cell Ther. (2014) 7:157–161. doi: 10.1016/j.hemonc.2014.06.005, PMID: 25066795

[5] Hoang-Xuan K Bessell E Bromberg J Hottinger AF Preusser M Ruda R . Diagnosis and treatment of primary CNS lymphoma in immunocompetent patients: guidelines from the European Association for Neuro-Oncology. Lancet Oncol. (2015) 16:e322–e332. doi: 10.1016/S1470-2045(15)00076-5, PMID: 26149884

[6] Shenkier TN Blay JY O’Neill BP Poortmans P Thele E Jahnke K . Primary CNS lymphoma of T-cell origin: a descriptive analysis from the international primary CNS lymphoma collaborative group. J Clin Oncol. (2005) 23:2233–2239. doi: 10.1200/JCO.2005.07.109, PMID: 15800313

[7] Chihara D Dunleavy K . Primary central nervous system lymphoma: evolving biologic insights and recent therapeutic advances. Clin Lymphoma Myeloma Leuk. (2021) 21:73–79. doi: 10.1016/j.clml.2020.10.015, PMID: 33288483

[8] Tringale KR Scordo M Yahalom J White C Zhang Z Vachha B . Outcomes and relapse patterns in primary central nervous system lymphoma: Longitudinal analysis of 559 patients diagnosed from 1983 to 2020. Neuro Oncol. (2024) 26:2061–2073. doi: 10.1093/neuonc/noae115, PMID: 38915246 PMC11534311

[9] Zhao E Yang YF Bai M Zhang H Yang YY Song X . MRI radiomics-based interpretable model and nomogram for preoperative prediction of Ki-67 expression status in primary central nervous system lymphoma. Front Med (Lausanne). (2024) 11:1345162. doi: 10.3389/fmed.2024.1345162, PMID: 38994341 PMC11236568

[10] Hoang-Xuan K Deckert M Ferreri AJM Further J Gallego Perez-Larraga J Henriksson R . European Association of Neuro-Oncology (EANO) guidelines for treatment of primary central nervous system lymphoma (PCNSL). Neuro Oncol. (2023) 25:37–53. doi: 10.1093/neuonc/noac196, PMID: 35953526 PMC9825335

[11] Alaggio R Amador C Anagnostopoulos I Artyagale AD Araujo BO Berti E . The 5th edition of the world health organization classification of haematolymphoid tumours: lymphoid neoplasms. Leukemia. (2022) 36:1720–1748. doi: 10.1038/s41375-022-01620-2, PMID: 35732829 PMC9214472

[12] Ferreri AJM Illerhaus G Doorduijn JK Auer DP Bromberg JE Calimeri T . Primary central nervous system lymphomas: EHA-ESMO Clinical Practice Guideline for diagnosis, treatment and follow-up. Hemasphere. (2024) 8:e89. doi: 10.1002/hem3.89, PMID: 38836097 PMC11148853

[13] Hida T . Anaplastic lymphoma kinase inhibitor development: enhanced delivery to the central nervous system. Transl Lung Cancer Res. (2023) 12:1822–1825. doi: 10.21037/tlcr-23-43, PMID: 37691872 PMC10483074

[14] Zhou X Wang F Yu L Yang F Kang J Cao D . Prediction of PD-L1 and Ki-67 status in primary central nervous system diffuse large B-cell lymphoma by diffusion and perfusion MRI: a preliminary study. BMC Med Imaging. (2024) 24:222. doi: 10.1186/s12880-024-01409-y, PMID: 39187807 PMC11348779

