# Supplementary figures and images for: Case Report: Cerebellar ALK+ ALCL: diagnostic challenges and therapeutic innovation
Source: Front Oncol. 2026 Mar 25;16:1620819. doi: 10.3389/fonc.2026.1620819 (PMC13056840; doi:10.3389/fonc.2026.1620819)

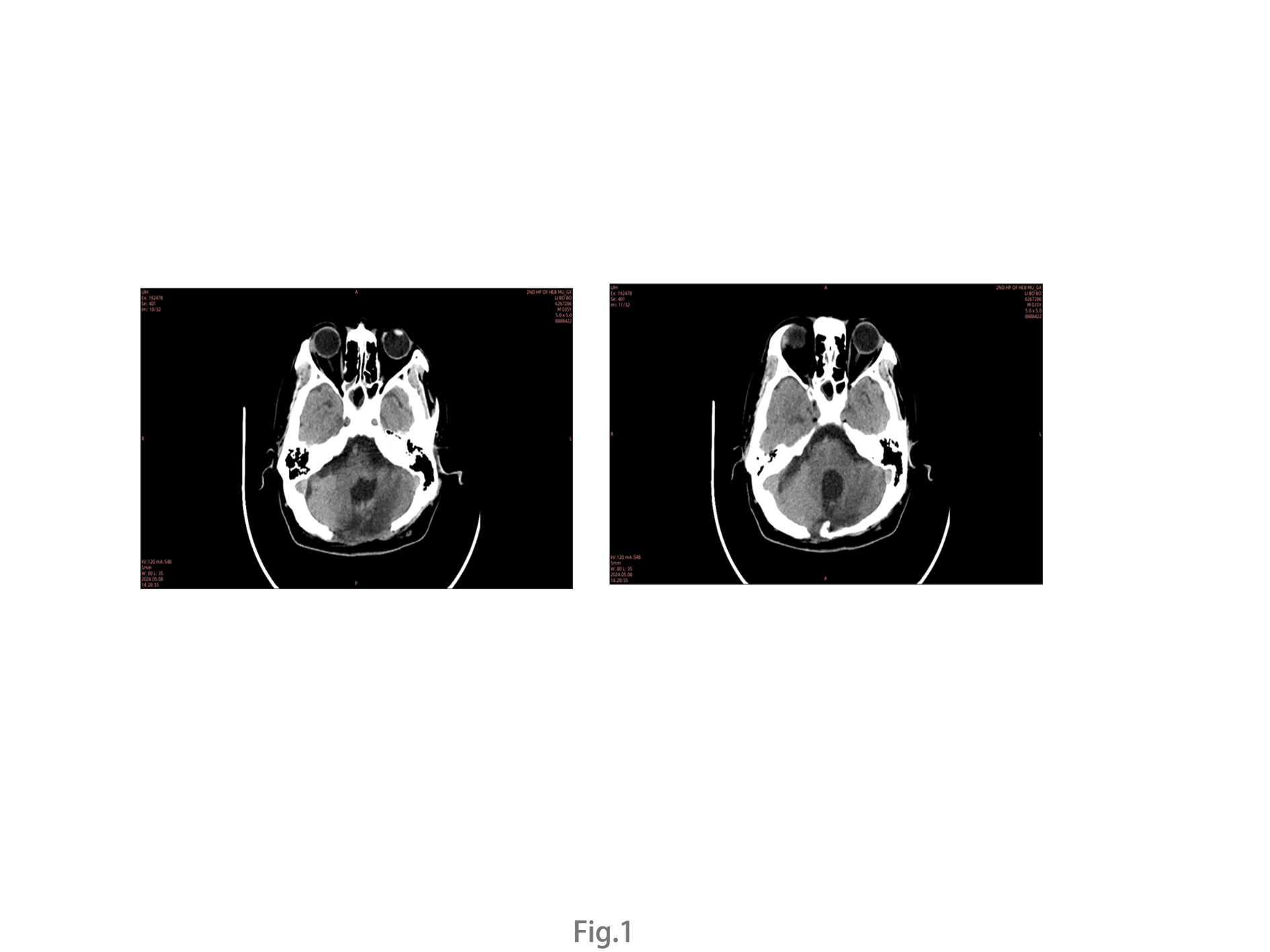

Supplement: Supplementary file 1 [file Image1.tif]
